# Conjunctival pigmented lesion: Clinicopathological analysis of 85 cases in Korean population

**DOI:** 10.1038/s41598-019-54786-8

**Published:** 2019-12-03

**Authors:** Yun Jeong Lee, Cheol Lee, Mee Kum Kim, Sang In Khwarg, Joo Youn Oh

**Affiliations:** 1 Department of Ophthalmology, Seoul National University College of Medicine, 103 Daehak-ro, Jongno-gu, Seoul 03080 Korea; 2 Department of Pathology, Seoul National University College of Medicine, 103 Daehak-ro, Jongno-gu, Seoul 03080 Korea; 3 Laboratory of Ocular Regenerative Medicine and Immunology, Biomedical Research Institute, Seoul National University Hospital, 101 Daehak-ro, Jongno-gu, Seoul 03080 Korea

**Keywords:** Conjunctival diseases, Outcomes research

## Abstract

To evaluate histopathological characteristics of conjunctival pigmented lesions and analyze clinical features related to histologic classification in Asian population, we analyzed medical records, anterior segment photographs, and histological specimen of 85 eyes who had undergone biopsy for pigmented conjunctival lesions at Seoul National University Hospital between 1999 and 2018. Compound nevus was the most common type of conjunctival pigmented lesions (67.1%), followed by conjunctival melanocytic intraepithelial neoplasia (primary acquired melanosis)(11.8%), subepithelial nevus (8.2%), and malignant melanoma (MM)(7.1%). Patients with compound nevus were younger than those with non-compound nevus (22.1 ± 17.0 vs 39.9 ± 18.8 years, *p* < 0.001), while patients with MM were older than those without melanoma (55.7 ± 18.2 vs 25.8 ± 18.0 years, p = 0.001). The lesion in compound nevus tended to be more frequently located on the temporal conjunctiva than that in the non-compound nevus group (54.4% vs 32.1%, *p* = 0.053), and feeder vessels were associated with most of compound nevus (98.2% vs 78.6% of non-compound nevus, *p* = 0.005). The lesion in MM was larger, involved multiple quadrants, and had extrabulbar location than lesions without melanoma (*p* < 0.001, *p* < 0.001, and *p* = 0.002, respectively). Together, the results would help clinicians to distinguish benign conjunctival pigmentations from malignant counterparts in clinical practice without biopsy.

## Introduction

Conjunctival melanocytic lesions are the most common tumors of the conjunctiva, comprising 52% of all conjunctival tumors^1–3^. Several classifications have been suggested for conjunctival melanocytic lesions^4^. Thus far, the 1980 World Health Organization (WHO) classification has been most widely used and includes three pathologic categories: conjunctival nevus, conjunctival melanosis, and malignant melanoma (MM)^5^. Conjunctival nevus is further histologically classified into three types by its location relative to the surface epithelium: compound, junctional, and subepithelial nevus^5^. Recently, IARC (International Agency for Research on Cancer) introduced the 2018 WHO classification of tumours of the eye^6^. Based on this new classification, melanocytic tumors of the conjunctiva are categorized into conjunctival nevus (junctional, compound, and subepithelial), conjunctival melanocytic intraepithelial neoplasia (C-MIN) that include primary acquired melanosis (PAM) with and without atypia, conjunctival melanoma, and others (benign epithelial melanoses of the conjunctiva, inflamed juvenile conjunctival nevus, blue nevus, Spitz or spindle cell nevus)^6^.

The compound nevus is the most common type of conjunctival melanocytic tumors and has a benign course which does not require treatment^5–8^. On the contrary, conjunctival MM is rare and carries serious consequences such as destruction of ocular tissues, distant metastasis (25%), and mortality (13–30%)^9–13^.

Given these wide variations in disease course, clinical distinction among conjunctival melanocytic lesions is essential to determine appropriate management of the lesion^14^. Importantly, the prevalence and prognosis of nevus and melanoma vary considerably among different racial/ethnic groups and geographic regions^15,16^. However, most of studies on conjunctival melanocytic lesions have been performed in Caucasian populations. Therefore, we herein performed a retrospective case-series study to report the prevalence of each histological type of conjunctival pigmented lesions in Asian (Korean) population according to the new 2018 WHO classification and to analyze demographic and clinical characteristics associated with each histologic classification.

## Results

### Demographics and disease laterality

Medical records, ocular anterior segment photographs, and histopathological specimens were reviewed of patients who visited Seoul National University Hospital for evaluation of conjunctival pigmented lesions between 1999 and 2018. During the study period, a total of 149 patients with conjunctival pigmented lesions visited our hospital. Among them, 64 patients were excluded from the study because of the following reasons: 46 patients did not undergo biopsy, 6 had an insufficient specimen for histologic assessment, 2 were Caucasians (non-Asian ethnicity), and in 10 patients, the pre-operative anterior segment photographs were unavailable for review of clinical characteristics of lesions. Consequently, a total of 85 eyes fit the inclusion criteria and were analyzed for the study. Patient demographics and disease laterality were summarized in Table 1. All patients were Korean by ethnicity and comprised 38 male and 47 female patients. The mean patient age was 27.1 ± 19.8 years (range 1–78 years) at the time of clinical diagnosis and 27.9 ± 19.5 years (range 4–78) at the time of histological diagnosis. The lesion involved the right eye in 37 eyes and left eye in 48 eyes.

**Table 1:** Patient demographics, histological classification of conjunctival pigmented lesions, and their association.

	Total	Compound nevus (n = 57)	Subepithelial nevus (n = 7)	C-MIN^a^/PAM^b^ (n = 10)					Malignant melanoma (n = 6)		Others^c^ (n = 5)
				PAM with atypia	PAM without atypia	C-MIN score			de novo	associated with nevus	
						1	2	4			
No. eyes (%)	85 (100.0)	57 (67.1)	7 (8.2)	1 (1.2)	9 (10.6)	5 (5.9)	4 (4.7)	1 (1.2)	3 (3.5)	3 (3.5)	5 (5.9)
Sex (male:female)	85 (38:47)	57 (23:34)	7 (5:2)	1 (1:0)	9 (5:4)	5 (2:3)	4 (3:1)	1 (1:0)	3 (1:2)	3 (1:2)	5 (2:3)
Age at clinical diagnosis, year (Mean ± SD, range)	27.1 ± 19.8 (1–78)	21.0 ± 17.5 (1–73)	39.0 ± 12.6 (16–52)	56.0 ± 0.0 (56–56)	33.7 ± 13.3 (19–61)	35.0 ± 7.9 (29–48)	32.0 ± 19.5 (19–61)	56.0 ± 0.0 (56–56)	54.3 ± 21.5 (33–76)	56.7 ± 19.1 (41–78)	29.0 ± 25.8 (7–73)
Age at histological diagnosis, year (Mean ± SD, range)	27.9 ± 19.5 (4–78)	22.1 ± 17.0 (4–73)	39.6 ± 13.3 (16–54)	56.0 ± 0.0 (56–56)	33.9 ± 13.9 (19–62)	35.2 ± 8.9 (29–50)	32.3 ± 20.0 (19–62)	56.0 ± 0.0 (56–56)	54.3 ± 21.5 (33–76)	57.0 ± 19.0 (41–78)	29.2 ± 25.8 (7–73)
Laterality (right:left)	85 (37:48)	57 (25:32)	7 (2:5)	1 (0:1)	9 (6:3)	5 (3:2)	4 (3:1)	1 (0:1)	3 (1:2)	3 (0:3)	5 (3:2)
		**Compound nevus**		**Non-compound nevus**		***P*** **value**	**Compound nevus**		**C-MIN/PAM**		***P*** **value**
Age at clinical diagnosis, year (Mean ± SD, range)		21.0 ± 17.5 (1–73)		39.6 ± 18.6 (7–78)		<0.001^d^	21.0 ± 17.5 (1–73)		35.9 ± 14.4 (19–61)		0.003^d^
Age at histological diagnosis, year (Mean ± SD, range)		22.1 ± 17.0 (4–73)		39.9 ± 18.8 (7–78)		<0.001^d^	22.1 ± 17.0 (4–73)		36.1 ± 14.8 (19–62)		0.004^d^
		**Malignant melanoma**		**Non-malignant melanoma**		***P*** **value**	**Malignant melanoma**		**Compound nevus**		***P*** **value**
Age at clinical diagnosis, year (Mean ± SD, range)		55.5 ± 18.3 (33–78)		25.0 ± 18.3 (1–73)		<0.001^d^	55.5 ± 18.3 (33–78)		21.0 ± 17.5 (1–73)		<0.001^d^
Age at histological diagnosis, year (Mean ± SD, range)		55.7 ± 18.2 (33–78)		25.8 ± 18.0 (4–73)		0.001^d^	55.7 ± 18.2 (33–78)		22.1 ± 17.0 (4–73)		<0.001^d^

^a^C-MIN: Conjunctival melanocytic intraepithelial neoplasia; ^b^PAM: primary acquired melanosis; ^c^Others: junctional nevus (n = 1), benign epithelial melanoses (n = 1), spindle cell nevus (n = 1), and non-melanocytic lesions (n = 2); ^d^Mann-Whitney test.

### Histopathologic classification

**Figure 1:**
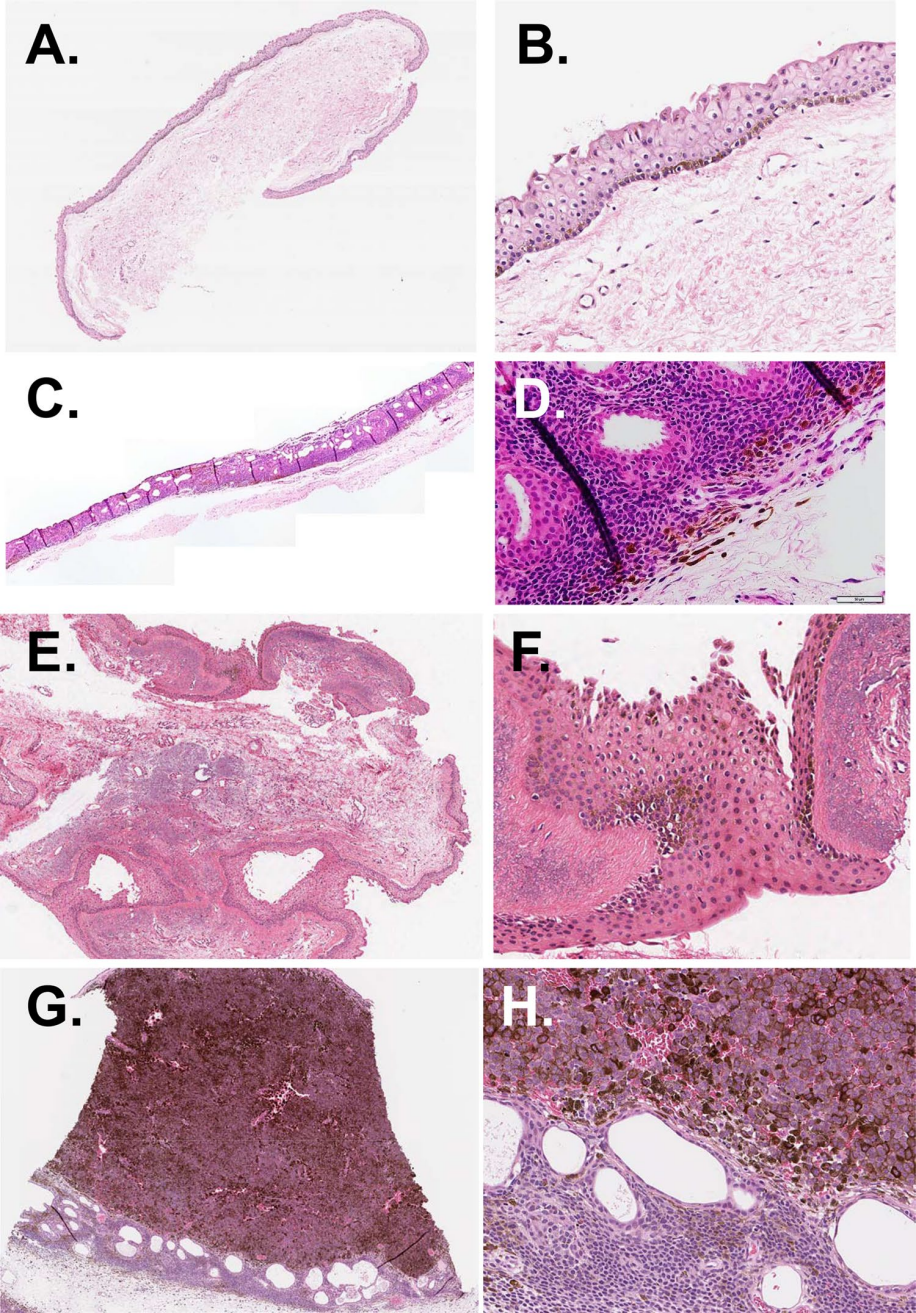
Representative photomicrographs of conjunctival pigmented lesions according to histological classification. **(A**,**B)** Benign epithelial melanoses of the conjunctiva. **(C**,**D)** Compound nevus. **(E**,**F)** Primary acquired melanosis without atypia. **(G**,**H)** Malignant melanoma. **(A,C,E,G)** Original magnification ×40. **(B,D,F,H)** Original magnification ×200. Scale bar 50 μm.

Histologic classification of the excised conjunctival pigmented lesions were shown in Table 1 and Fig. 1. Of 85 histopathologic specimens, compound nevus was the most common histologic diagnosis and accounted for 67.1% (57 eyes) of conjunctival pigmented lesions (Table 1). C-MIN/PAM were found in 11.8% (10 eyes) including PAM without atypia (9 eyes) and PAM with atypia (one eye). C-MIN scores were 1 in 5 eyes, 2 in 4 eyes, and 4 in one eye. Subepithelial nevus was found in 7 eyes (8.2%), and MM in 6 eyes (7.1%) including MM de novo (3 eyes) and MM associated with nevus (3 eyes). The remaining 5 eyes were diagnosed of having junctional nevus (one eye), benign epithelial melanoses (one eye), spindle cell nevus (one eye), and non-melanocytic lesions (2 eyes).

### Association between histologic subtype and age

Among histologic subtypes, patients with compound nevus were the youngest (22.1 ± 17.0 years, range 4–73 years), followed by C-MIN/PAM (36.1 ± 14.8 years, range 19–62 years), subepithelial nevus (39.6 ± 13.3 years, range 16–54), and MM (55.7 ± 18.2 years, range 33–78) (Table 1). A comparison between patients with compound nevus and non-compound nevus showed that the age was significantly younger in patients with compound nevus (*p* < 0.001 for both clinical and histological diagnosis age) (Table 1). Specifically, patients with compound nevus were significantly younger than patients with C-MIN/PAM (*p* = 0.003 for clinical diagnosis age and p = 0.004 for histological diagnosis age) or those with MM (*p* < 0.001 for both histological and clinical diagnosis age) (Table 1). By contrast, patients in the MM group were significantly older compared to those with non-MM lesions (*p* < 0.001 for clinical diagnosis age and *p* = 0.001 for histological diagnosis age) (Table 1). The disease laterality did not show significant difference between histologic types.

### Clinical characteristics and association with histologic subtype

**Figure 2:**
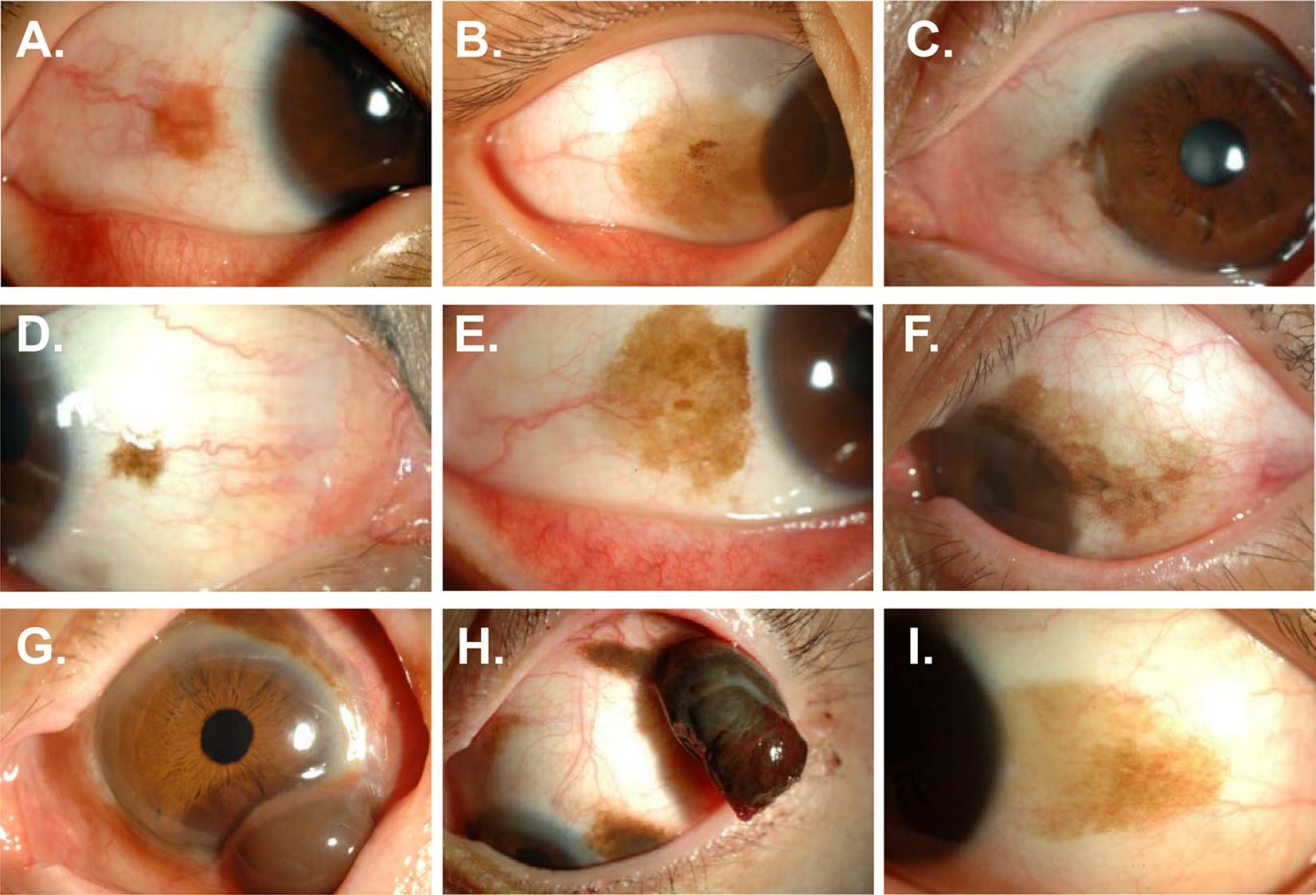
Representative anterior segment photographs of conjunctival pigmented lesions according to histological classification. **(A)** Compound nevus. **(B)** Junctional nevus. **(C)** Subepithelial nevus. **(D,E)** Primary acquired melanosis without atypia. **(F)** Primary acquired melanosis with atypia. **(G,H)** Malignant melanoma. **(I)** Benign epithelial melanoses of the conjunctiva.

Clinical characteristics of the lesion with respect to the location, size, and combined ocular abnormalities in each histologic classification were presented in Table 2 and Fig. 2. Additionally, clinical features of a lesion were compared among histologic subtypes for identification of clinical parameter(s) associated with a specific histologic diagnosis (Table 3).

**Table 2:** Clinical ocular characteristics of conjunctival pigmented lesions according to histological classification.

	Total	Compound nevus (n = 57)	Subepithelial nevus (n = 7)	C-MIN^a^/PAM^b^ (n = 10)		Malignant melanoma (n = 6)		Others^c^ (n = 5)
				PAM with atypia	PAM without atypia	de novo	associated with nevus	
Location, No. eyes (%)								
Superior	2 (2.4)	2 (3.5)	0 (0.0)	0 (0.0)	0 (0.0)	0 (0.0)	0 (0.0)	0 (0.0)
Nasal	24 (28.2)	12 (21.1)	7 (100.0)	0 (0.0)	5 (55.6)	0 (0.0)	0 (0.0)	0 (0.0)
Inferior	1 (1.2)	1 (1.8)	0 (0.0)	0 (0.0)	0 (0.0)	0 (0.0)	0 (0.0)	0 (0.0)
Temporal	42 (49.4)	33 (57.9)	0 (0.0)	0 (0.0)	4 (44.4)	0 (0.0)	1 (33.3)	4 (80.0)
Multiple	16 (18.8)	9 (15.8)	0 (0.0)	1 (100.0)	0 (0.0)	3 (100.0)	2 (66.7)	1 (20.0)
Location, No. eyes (%)								
Bulbar conjunctiva	65 (76.5)	47 (82.5)	3 (42.9)	1 (100.0)	9 (100.0)	0 (0.0)	1 (33.3)	4 (80.0)
Caruncle	7 (8.2)	3 (5.3)	4 (57.1)	0 (0.0)	0 (0.0)	0 (0.0)	0 (0.0)	0 (0.0)
Tarsal conjunctiva	0 (0.0)	0 (0.0)	0 (0.0)	0 (0.0)	0 (0.0)	0 (0.0)	0 (0.0)	0 (0.0)
Fornix	1 (1.2)	1 (1.8)	0 (0.0)	0 (0.0)	0 (0.0)	0 (0.0)	0 (0.0)	0 (0.0)
Multiple	12 (14.1)	6 (10.5)	0 (0.0)	0 (0.0)	0 (0.0)	3 (100.0)	2 (66.7)	1 (20.0)
Range (%)								
1 Quadrant	33 (38.8)	23 (40.4)	5 (71.4)	0 (0.0)	2 (22.2)	0 (0.0)	0 (0.0)	3 (60.0)
2 Quadrants	38 (44.7)	27 (47.4)	2 (28.6)	0 (0.0)	7 (77.8)	0 (0.0)	1 (33.3)	1 (20.0)
3 Quadrants	8 (9.4)	6 (10.5)	0 (0.0)	1 (100.0)	0 (0.0)	0 (0.0)	1 (33.3)	0 (0.0)
4 Quadrants	6 (7.1)	1 (1.8)	0 (0.0)	0 (0.0)	0 (0.0)	3 (100.0)	1 (33.3)	1 (20.0)
Size, mm (Mean ± SD, range)	7.4 ± 6.6 (1.5–42.0)	7.2 ± 6.8 (1.5–42.0)	3.5 ± 1.2 (2.0–5.0)	14.0 ± 0.0 (14.0–14.0)	5.4 ± 1.7 (3.0–8.0)	19.7 ± 4.5 (15.0–24.0)	14.3 ± 8.4 (9.0–24.0)	5.9 ± 4.3 (1.5–13.0)
Distance from limbus, mm (Mean ± SD, range)	1.4 ± 2.3 (0.0–12.0)	1.4 ± 1.9 (0.0–8.0)	4.0 ± 4.5 (0.0–12.0)	0.0 ± 0.0 (0.0–0.0)	0.8 ± 2.0 (0.0–6.0)	0.0 ± 0.0 (0.0 ± 0.0)	0.2 ± 0.3 (0.0–0.5)	0.2 ± 0.4 (0.0–1.0)
Corneal or pupil pigmentation, No. eyes (%)	30 (35.3)	22 (38.6)	1 (14.3)	1 (100.0)	2 (22.2)	2 (66.7)	1 (33.3)	1 (20.0)
Feeder vessel, No. eyes (%)	78 (91.8)	56 (98.2)	5 (71.4)	1 (100.0)	8 (88.9)	1 (33.3)	2 (66.7)	5 (100.0)

^a^C-MIN: Conjunctival melanocytic intraepithelial neoplasia; ^b^PAM: primary acquired melanosis; ^c^Others: junctional nevus (n = 1), benign epithelial melanoses (n = 1), spindle cell nevus (n = 1), and non-melanocytic lesions (n = 2).

**Table 3:** Comparison of clinical and ocular features among different histologic subtypes of conjunctival pigmented lesions.

	Compound nevus	Non-compound nevus	*P* value
Location, No. eyes (%)			
Superior	2 (3.5)	0 (0.0)	0.069^a^
Nasal	12 (21.1)	12 (42.9)	
Inferior	1 (1.8)	0 (0.0)	
Temporal	33 (57.9)	9 (32.1)	
Multiple	9 (15.8)	7 (25.0)	
Location, No. eyes (%)			
Bulbar conjunctiva	47 (82.5)	18 (64.3)	0.163^a^
Caruncle	3 (5.3)	4 (14.3)	
Tarsal conjunctiva	0 (0.0)	0 (0.0)	
Fornix	1 (1.8)	0 (0.0)	
Multiple	6 (10.5)	6 (21.4)	
Location, No. eyes (%)			
Temporal conjunctiva	31 (54.4)	9 (32.1)	0.053^b^
Temporal bulbar conjunctiva	29 (50.9)	9 (32.1)	0.103^b^
Feeder vessel, No. eyes (%)	56 (98.2)	22 (78.6)	0.005^a^
	**Malignant melanoma**	**Non-malignant melanoma**	***P*** **value**
Location, No. eyes (%)			
Bulbar conjunctiva	1 (16.7)	64 (81.0)	0.002^a^
Extra-bulbar conjunctiva	5 (83.3)	15 (19.0)	
Range, No. eyes (%)			
1 or 2 Quadrant(s)	1 (16.7)	70 (88.6)	<0.001^a^
3 or 4 Quadrants	5 (83.3)	9 (11.4)	
Size, mm (Mean ± SD, range)	17.0 ± 6.7 (9.0–24.0)	6.7 ± 6.0 (1.5–42.0)	<0.001^c^
	**Malignant melanoma**	**Compound nevus**	***P*** **value**
Location, No. eyes (%)			
Bulbar conjunctiva	1 (16.7)	47 (82.5)	0.002^a^
Extra-bulbar conjunctiva	5 (83.3)	10 (17.5)	
Range, No. eyes (%)			
1 or 2 Quadrant(s)	1 (16.7)	50 (87.7)	0.001^a^
3 or 4 Quadrants	5 (83.3)	7 (12.3)	
Size, mm (Mean ± SD, range)	17.0 ± 6.7 (9.0–24.0)	7.2 ± 6.8 (1.5–42.0)	<0.001^c^
	**C-MIN**^d^	**Compound nevus**	***P*** **value**
Location, No. eyes (%)			
Bulbar conjunctiva	10 (100.0)	47 (82.5)	0.337^a^
Extra-bulbar conjunctiva	0 (0.0)	10 (17.5)	
Range, No. eyes (%)			
1 or 2 Quadrant(s)	9 (90.0)	50 (87.7)	1.000^a^
3 or 4 Quadrants	1 (10.0)	7 (12.3)	
Size, mm (Mean ± SD, range)	6.3 ± 3.2 (3.0–14.0)	7.2 ± 6.8 (1.5–42.0)	0.804^c^

^a^Fisher’s exact test; ^b^Chi-square test; ^c^Mann-Whitney test; ^d^C-MIN: Conjunctival melanocytic intraepithelial neoplasia.

Overall, 76.5% (65 eyes) of conjunctival pigmented lesions were located on the bulbar conjunctiva, 8.2% (7 eyes) on the caruncle, and 1.2% (one eye) in the fornix (Table 2). Twelve eyes (14.1%) involved multiple sites. An analysis by histologic subtype showed that the majority of compound nevus (82.5%) was located on the bulbar conjunctiva. In contrast, only 42.9% of subepithelial nevus was bulbar and the remaining 57.1% of subepithelial nevus was located on the caruncle. Five of 6 eyes with MM (83.3%) had diffuse extrabulbar locations. The comparison between MM and non-MM revealed that the extrabulbar location of pigmentation was more common in eyes with MM than in those with the non-MM lesions (83.3% vs 19.0%, *p* = 0.002) (Table 3). Also, comparison between MM and compound nevus showed that the lesion in MM involved the extrabulbar area more commonly than in the compound nevus (83.3% vs 17.5%, *p* = 0.002) (Table 3).

The location of a lesion was further analyzed by quadrants. Totally, 49.4% (42 eyes) of conjunctival pigmented lesions were located at the temporal quadrant, 28.2% (24 eyes) at the nasal quadrant, 2.4% (2 eyes) at the superior quadrant, and 1.2% (one eye) at the inferior quadrant (Table 2). Multiple quadrants were affected in 16 eyes (18.8%). When each histologic subtype was analyzed by quadrant location, 57.9% of compound nevus was located at the temporal quadrant, while 100% of subepithelial nevus was located at the nasal quadrant (Table 2). A comparison of the compound nevus vs non-compound nevus revealed that the lesion tended to be more frequently located on the temporal conjunctiva in eyes with compound nevus than in eyes with the non-compound nevus with a borderline significance (*p* = 0.053) (Table 3).

The size of a lesion was measured either by the longest diameter or by the number of quadrant affected by pigmentation. The average diameter of conjunctival pigmented lesion was 7.4 ± 6.6 mm (range 1.5–42.0 mm), and most of lesions (83.5%) involved one or two quadrants (Table 2). Further analysis of the lesion size according to a specific histologic type showed that the size of lesion was the largest in MM (17.0 ± 6.7 mm, range 9.0–24.0 mm) (Table 2) and significantly larger in MM, compared with the non-MM groups (17.0 ± 6.7 mm vs. 6.7 ± 6.0 mm, *p* < 0.001) or with compound nevus (17.0 ± 6.7 mm vs. 7.2 ± 6.8 mm, *p* < 0.001) (Table 3). Also, the lesion involved more than two quadrants in 5 of 6 eyes with MM (83.3%) (3 quadrants in one eye and all 4 quadrants in 4 eyes), whereas the majority of conjunctival nevus (87.7% of compound nevus and 100% of subepithelial nevus) affected one or two quadrants (Table 2). Differences in the number of quadrants involved were statistically significant between MM vs non-MM lesions (*p* < 0.001) and between MM vs compound nevus groups (*p* = 0.001) (Table 3). However, there were no significant differences in the location and size between C-MIN/PAM and compound nevus (Table 3).

Distance of the pigmented lesion from limbus was 1.4 mm on average with being the longest in subepithelial nevus (4.0 ± 4.5 mm) and shortest in MM (0.1 ± 0.2 mm) (Table 2); however, the differences were not significant between subepithelial and non-subepithelial nevi or between MM and non-MM nevi (*p* = 0.114 and 0.096, respectively).

Pigmentation was also present in the cornea or iris in 35.3% of cases (30 of 85 eyes), and 91.8% of pigmented lesions (78 eyes) were accompanied by feeder vessels (Table 2). In particular, feeder vessels were more commonly associated with the compound nevus, compared with the non-compound nevus (98.2% vs. 78.6%, *p* = 0.005) (Table 3).

## Discussion

To summarize, we found that the compound nevus was identified as the most common conjunctival pigmented lesion (67.1%) in Korean population, and MM was rare (7.1%). We additionally correlated demographic and clinical features with histologic diagnosis. Based on demographics, the mean age at diagnosis was younger in patients with compound and junctional nevi (22.1 and 7.0 years, respectively), compared with subepithelial nevus, C-MIN/PAM, and MM (39.6, 36.1, and 55.7 years, respectively). Based on clinical ocular characteristics, MM was differentiated from its benign counterparts by its larger size, involvement of multiple conjunctival quadrants, and extrabulbar location (Fig. 2G,H). In contrast, the compound nevus often appeared on the temporal conjunctiva, affecting one or two quadrants and accompanying feeder vessels (Fig. 2A).

Our results are generally in line with other reports. The compound nevus was the most prevalent among conjunctival nevus, followed by C-MIN/PAM and subepithelial nevus^5,7,17^. Compound nevus was found in younger patients, whereas subepithelial nevus was more frequent in older age groups^5,7^. These findings might be explained by the natural cycle of nevus cells that form a group of small nests of pigment epithelial cells in the basal layer of the epithelium in early stage and move deeper into the underlying stroma to become the compound nevus with age^18^. As further migration occurs, nevus cells tend to lose pigments and reside in the stroma as sub-epithelial nevus during the third and fourth decades. In our case series, junctional nevus was found in only one 7-year-old female patient. The paucity of junctional nevus in our study might be attributed to the fact that junctional nevus occurs in young age, appears clinically flat and focal without dense pigmentation or feeder vessels, and rarely aggravates^6^; therefore, biopsy is rarely performed.

One notable finding of our study is that the prevalence of C-MIN/PAM and MM was low in our population, accounting for 11.8% and 7.1%, respectively. By comparison, previous studies performed in USA demonstrated that among biopsied conjunctival melanocytic lesions, the diagnosis was PAM in 21.0–24.0% and MM in 23.2–25.0%^1,2^. Also, another study by Shields *et al*. reported that MM was found in 18 (2.2%) of 510 conjunctival pigmented tumor patients younger than 21 years^3^. By contrast, there was no MM case in young patients in our study, and the youngest age at the time of MM diagnosis was 33 years. Consistent with our finding, other case series of MM patients in Chinese and Korean populations showed the low incidence of the tumor in young patients^16,20^. These findings collectively indicate the racial/ethnic difference in the development of MM between Asian and non-Asian populations because all 18 young patients diagnosed of MM in the study by Shields *et al*. were whites (89%) or African Americans (11%)^3^. Otherwise, our MM cases share common clinical features such as older patient age, larger base diameter, more frequent extrabulbar location with MM cases in other reports^2,3,16,19,20^.

Our study is a cross-sectional, retrospective case-series, and therefore, the natural course of disease or post-surgical outcome could not be evaluated. Also, a small number of MM in our study precluded determining whether the features associated with malignancy were independent of one another. Future studies in a larger number of patients over the longer follow-up period would be necessary to investigate the course and prognosis of each conjunctival pigmented tumor and identify clinical characteristics associated with each tumor. Despite these limitations, our study has strength because it depicted histopathologically-confirmed cases of conjunctival pigmented lesions in Asian population where most of lesions are considered benign and thus managed largely by observation or laser photocoagulation without undergoing biopsy^21^.

In conclusion, the most common histopathologic diagnosis of conjunctival pigmented lesions in Korean population was the compound nevus. MM was rare and could be differentiated from benign pigmented tumors by older age of patient, larger diameter of lesion, multiple involvement of conjunctival quadrants, and extrabulbar location. Our results would be beneficial for clinicians in distinguishing benign conjunctival pigmentations from malignant counterparts in clinical practice without excision and biopsy.

## Methods

This retrospective case-series study was approved by the Institutional Review Board of Seoul National University Hospital (IRB No. 1904–095–1027) and was carried out in accordance with the Declaration of Helsinki. The informed consent was obtained prior to excisional biopsy.

Medical records, ocular anterior segment photographs, and histopathological specimens were reviewed of patients who had undergone excisional biopsy for pigmented conjunctival lesions at Seoul National University Hospital between 1999 and 2018. Excluded were the conjunctival melanocytic lesions which were not confirmed by histology or the lesions without anterior segment photographs taken prior to biopsy. The lesions from non-Asian patients and histopathologic specimens insufficient for histologic classification were also excluded. The criteria for performing excisional biopsy included the suspicion for malignant changes based on clinical features of a lesion (color change, rapid growth, recurrence of a previously excised lesion, or involvement of multiple sites in the cornea, sclera, and fornix) and the patient’s motivation (concern of cancer, cosmetic reason, or ocular irritation). From medical records, demographic information (age at the time of clinical diagnosis, age at the time of histological diagnosis, sex) and laterality of a disease were collected. By the review of anterior segment photographs, clinical characteristics such as location and size of a lesion, distance of a lesion from limbus, the presence of combined pigmentation in the cornea or iris, or the presence of feeder vessels were analyzed. Histopathologic slides were reviewed by an ophthalmic pathologist (C.L.) specializing in ocular and skin tumors, and the lesions were histologically classified into the following categories as defined by the 2018 WHO classification of tumours of the eye^6^ (Fig. 1): conjunctival nevus (junctional, compound, and subepithelial), C-MIN including PAM with and without atypia, conjunctival MM (de novo or associated with a pre-existing nevus), and others (benign epithelial melanoses and spindle cell nevus). Immunohistochemical staining for HMB-45 antigen, S-100 protein, melan-A, Ki-67, and p53 was performed to help the diagnosis in cases where the differentiation between PAM without atypia and nevus was difficult.

For statistical analysis, descriptive statistics, Fisher’s exact test, Chi-square test, and Mann-Whitney test were used to compare quantitative and qualitative variables among groups. Analysis was performed using SPSS software (version 23.0, IBM Corp., Armonk, NY), and comparisons were regarded as significant for *p* < 0.05.
